# TRPV4-mediates oscillatory fluid shear mechanotransduction in mesenchymal stem cells in part via the primary cilium

**DOI:** 10.1038/s41598-018-22174-3

**Published:** 2018-02-28

**Authors:** Michele A. Corrigan, Gillian P. Johnson, Elena Stavenschi, Mathieu Riffault, Marie-Noelle Labour, David A. Hoey

**Affiliations:** 1 Trinity Centre for Bioengineering, Trinity Biomedical Sciences Institute, Trinity College Dublin, Dublin, 2 Ireland; 2 Department of Mechanical and Manufacturing Engineering, School of Engineering, Trinity College Dublin, Dublin, 2 Ireland; 3 Department of Mechanical, Aeronautical and Biomedical Engineering, University of Limerick, Limerick, Ireland; 4 Advanced Materials and Bioengineering Research Centre, Trinity College Dublin & RCSI, Dublin, 2 Ireland

## Abstract

Skeletal homeostasis requires the continued replenishment of the bone forming osteoblast from a mesenchymal stem cell (MSC) population, a process that has been shown to be mechanically regulated. However, the mechanisms by which a biophysical stimulus can induce a change in biochemical signaling, mechanotransduction, is poorly understood. As a precursor to loading-induced bone formation, deciphering the molecular mechanisms of MSC osteogenesis is a critical step in developing novel anabolic therapies. Therefore, in this study we characterize the expression of the mechanosensitive calcium channel Transient Receptor Potential subfamily V member 4 (TRPV4) in MSCs and demonstrate that TRPV4 localizes to areas of high strain, specifically the primary cilium. We demonstrate that TRPV4 is required for MSC mechanotransduction, mediating oscillatory fluid shear induced calcium signaling and early osteogenic gene expression. Furthermore, we demonstrate that TRPV4 can be activated pharmacologically eliciting a response that mirrors that seen with mechanical stimulation. Lastly, we show that TRPV4 localization to the primary cilium is functionally significant, with MSCs with defective primary cilia exhibiting an inhibited osteogenic response to TRPV4 activation. Collectively, this data demonstrates a novel mechanism of stem cell mechanotransduction, which can be targeted therapeutically, and further highlights the critical role of the primary cilium in MSC biology.

## Introduction

Skeletal homeostasis and repair requires the continued replenishment of the bone forming osteoblast from a mesenchymal stem cell (MSC) population^1^. This process of MSC differentiation has been shown to be mechanically regulated, with physical loading promoting MSC osteogenesis and bone formation^2,3^. However, the molecular mechanisms by which mechanical stimuli can induce a change in biochemical cellular signaling, termed mechanotransduction, is poorly understood. With aging and the onset of osteoporosis, the number and osteogenic potential of MSCs is diminished^4,5^, leading to a decoupling of the bone formation/resorption cycle and net bone loss^6^. Given the potent role of physical activity in regulating stem cell differentiation and bone formation, deciphering the mechanisms of mechanotransduction may provide novel targets to promote bone formation by mimicking the beneficial effects of loading at a molecular level^7^.

During loading-induced deformation of bone, MSCs within the marrow are predicted to experience a complex array of mechanical stimuli that includes oscillatory fluid flow induced shear stress^8^, which has been shown *in vitro* to drive MSC proliferation and differentiation^3,9,10^. Several studies have explored the role of fluid shear (FS) in driving osteogenic responses in MSCs, with varying magnitudes and durations of mechanical stimuli making direct comparisons challenging. Human MSCs (hMSCs) exposed to FS display an upregulation in bone morphogenetic protein 2 (*BMP2*), osteopontin (*OPN*)^11^ after 24hrs stimulation or runt related transcription factor (*RUNX2*), alkaline phosphatase (*ALPL*), and *OPN*^12^ after 48hrs stimulation. A recent systematic study on the effect of oscillatory fluid shear (OFS) variables such as shear magnitude and frequency has demonstrated an increase in Cyclo-oxygenase 2 (*Cox2*), *Opn* and *Runx2* expression at early time points, that over 21 days results in enhanced collagen and matrix mineralization^3^, which is an agreement with a number of studies demonstrating fluid shear induced MSC osteogenesis^13–15^. Cytosolic Ca^2+^ is a primary second messenger in the control and regulation of a wide range of stem cell functions and is released from intracellular stores in response to fluid shear, with cytosolic calcium levels peaking within seconds of the application of flow^10^. The role of this initial calcium signaling in triggering downstream osteogenic transcriptional activity is widely appreciated, as demonstrated in osteoblasts where removal of the calcium flux within the cytosol prevents the fluid shear mediated upregulation in *Opn*^16^. Furthermore, a loss of this calcium signal impairs the capacity of osteoprogenitors to proliferate and form mineralized tissue^17^. Despite the well characterized effect of OFS on calcium signaling and differentiation, the mechanisms by which MSCs sense this mechanical stimulus remains poorly understood.

The application of fluid shear to MSCs results in strain across the membrane that is concentrated at specific regions, such as focal adhesions and at the primary cilium^18^. These sites of maximum strain have been shown to be important for stem cell fluid shear mechanotransduction^13,19^. Focal adhesions are large macromolecular assemblies that are the initial site of interaction between the cell and the extracellular matrix (ECM), via integrin attachments. Integrins facilitate increased cytoskeletal interaction and provide binding sites for a multitude of effectors, activating key mechanosignaling pathways^20,21^. The primary cilium is a solitary cellular organelle that extends from the cell membrane^22,23^. Previous work in kidney epithelial cells and osteocytes suggests it is positioned apically and so exposed to fluid shear in the extracellular fluid between cells^24^. The cilium is composed of microtubules along which specialised motor proteins known as intraflagellar transport proteins function to transport cargo along the axoneme. Absence of a functional cilium including the IFT component IFT88 disrupts skeletal development^25,26^. Spatially removed from the cytoplasm, the ciliary microdomain is highly enriched in signaling molecules, receptors and ion channels^23,27,28^, and as such plays critical roles in stem cell function including mechanotransduction^13^. Interestingly, both focal adhesions and the primary cilium have been linked to mechanically-induced calcium signaling. Mechanical stretch of integrins and/or bending of the cilium leads to a rapid increase in local and cytosolic calcium levels^24,29^. Fluid shear induced strain has the capacity to open stretch-activated calcium channels, motivating the identification of these specific channels which mediate calcium signals during MSC mechanotransduction.

The Transient Receptor Potential (TRP) family is a group of calcium permeable membrane spanning ion channels. Defined in 7 subgroups, they display diverse stimulatory mechanisms including mechanical activation^30^. Substantial evidence exists for the role of TRP subfamily V member 4 (TRPV4) in mechanosensation. TRPV4 is required for loading induced responses in committed cells such as chondrocytes^31^, osteocytes^24^, epithelial^32^ and endothelial cells^33^. Furthermore, TRPV4 has been previously found to have a preferential spatial cellular organization to areas of high strain, such as at focal adhesions^29^ and along the primary cilium^24,34^. Mutations in TRPV4 have been linked to human skeletal pathologies^35^ and TRPV4 KO mice do not lose bone in a hind limb suspension model^36^, strongly indicating that TRPV4 plays a role in skeletal mechanobiology. Furthermore, MSCs isolated from TRPV4 KO mice demonstrate an inhibited osteogenic potential^37^. Although the role of TRPV4 in mechanotransduction in mature cells of the musculoskeletal system is well demonstrated, in MSCs its subcellular spatial organization and potential role in mechanotransduction are poorly characterized.

As a precursor to loading-induced bone formation, deciphering the molecular mechanisms of MSC osteogenesis is a critical step in developing novel anabolic therapies to promote bone regeneration. Therefore, in this study we characterize the expression of TRPV4 in MSCs and demonstrate that TRPV4 localizes to areas of high strain, specifically the primary cilium, and show that this mechanosensitive channel is required for stem cell mechanotransduction, mediating fluid shear induced calcium signaling and osteogenic gene expression. Furthermore, we demonstrate that TRPV4 can be activated pharmacologically eliciting an osteogenic response that mirrors both the flux in calcium signal and upregulation in early osteogenic genes activated by fluid shear, demonstrating mechanotherapy potential. Lastly, we show that TRPV4 localization to the primary cilium is functionally significant, with MSCs with defective primary cilia exhibiting an inhibited osteogenic response to TRPV4 activation. Collectively, this data demonstrates a novel mechanism of stem cell mechanotransduction, which can be targeted therapeutically, and further highlights the critical role of the primary cilium in stem cell biology.

## Results

### Mesenchymal stem cells respond to oscillatory fluid shear with a rapid increase in cytosolic calcium and osteogenic gene expression

**Figure 1:**
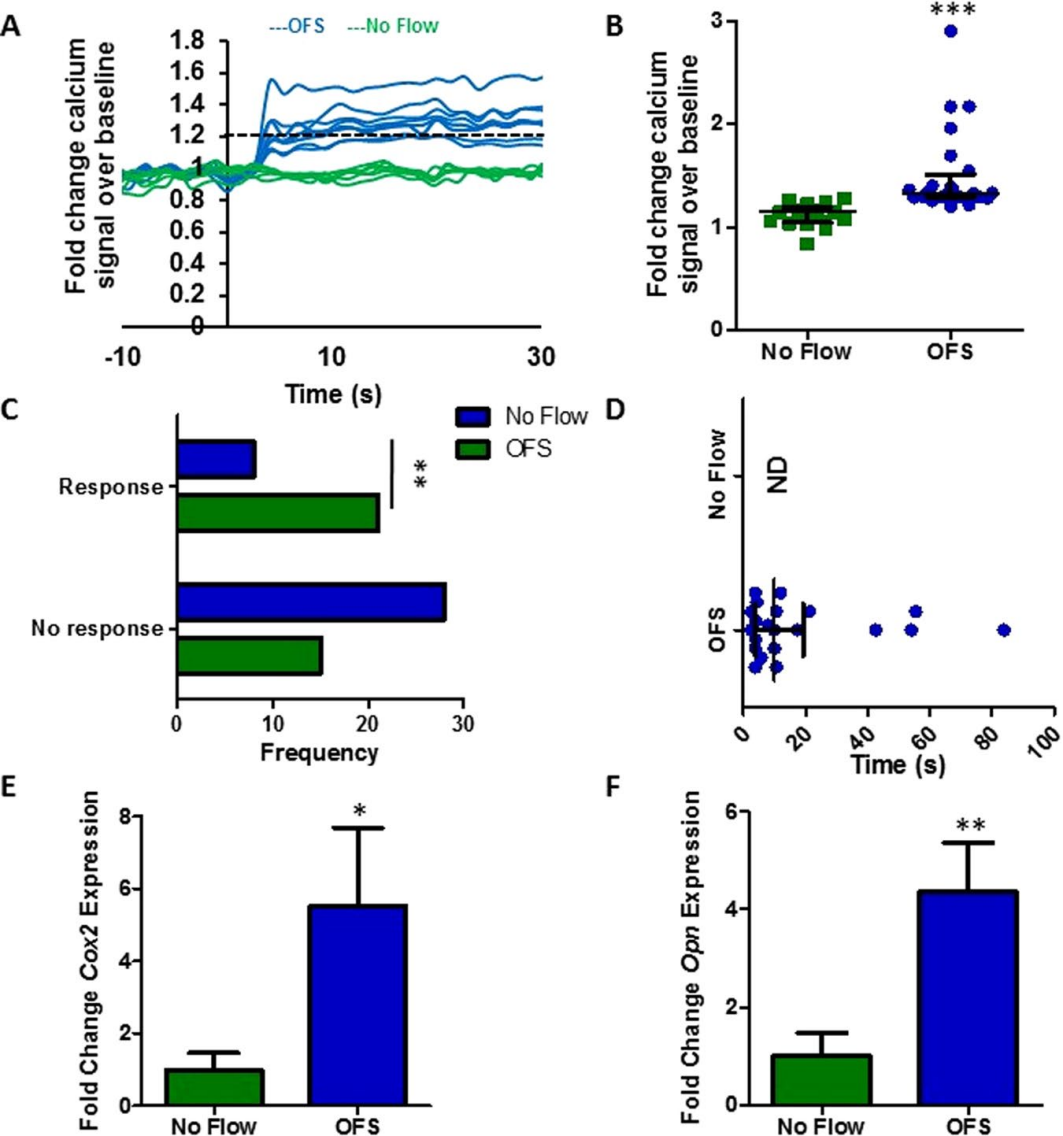
(**A**) Representative calcium profiles of individual MSCs during No Flow (green) and oscillatory fluid shear (OFS) generating 1 Pa, 1 Hz shear stress (blue). Stimulus begins at t = 0 s. Baseline values calculated from 20 seconds prior to the application of OFS. The dashed black line at 1.2 fold change marks the value above which cells are considered to be responsive. **(B)** Fold increase in calcium at first peak during No Flow and OFS, Median ± Interquartile, No Flow, n = 17; OFS, n = 21, Mann-Whitney test, ***p < 0.001. **(C)** Frequency of cells eliciting a response or showing no response in static (blue) and OFS (green) conditions, N = 3, n = 36, Fisher’s exact test, **p < 0.01. **(D)** Time from onset of flow (t = 0 s) to first peak, Median ± Interquartile range, NF n = 17, OFS n = 21. **(E**,**F)** Expression of early osteogenic markers in MSCs following application of 1 Pa, 1 Hz OFS for 2 hours, Mean ± SEM (**E**) *Cox2* N = 4, n = 8–13, **(F)** *Opn* N = 4, n = 14–20, student’s t-test, *p < 0.05, **p < 0.01.

To characterize the response of MSCs to fluid flow induced shear stress, OFS was applied at 1 Pa, 1 Hz, mimicking physiological mechanics predicted to occur within the bone marrow^13^. To determine the initial response to a mechanical stimulus, calcium levels within the cell were determined in real-time using a fluorescent indicator. MSCs were initially exposed to a laminar fluid flow-induced shear stress of 0.01 Pa for 2 mins to equilibrate the system. No change in intracellular calcium levels was detected during this period when compared to No Flow controls demonstrating that this low level of mechanical stimulus is insufficient to induce a mechanoresponse. MSCs were then exposed to OFS at 1 Pa and fold change fluorescence levels were quantified relative to no OFS. The application of 1 Pa OFS elicits a 1.53-fold increase in cytosolic calcium demonstrating that this increased mechanical stimulus is transduced by MSCs eliciting a second messenger signaling response (Fig. 1A,B). Taking a cell that demonstrates a calcium peak greater than 1.2-fold in response to OFS as mechanoresponsive, 1 Pa OFS induces a response in a significantly greater number of MSCs compared to static controls (Fig. 1C, p < 0.01). Furthermore, the response to OFS was immediate (17.64 ± 4.84 seconds) (Fig. 1D). To characterize a downstream response to mechanical stimuli, gene expression analysis was performed on cells exposed to 1 Pa OFS for 2 hours. In response to OFS, the expression of the osteogenic markers *Cox2* and *Opn* was significantly upregulated (5.52-fold, n ≥ 8, p < 0.05 and 4.35-fold, n ≥ 14, p < 0.01 respectively) compared to No Flow controls (Fig. 1E,F). Previous studies have shown that increases in *Cox2* and *Opn* are early markers of full osteogenic commitment in MSCs^3^. This data therefore demonstrates that OFS predicted to occur within the marrow elicits a positive mechanoresponse in MSCs in terms of second messenger calcium signaling and downstream osteogenic gene expression.

### TRPV4 is expressed by mesenchymal stem cells and is found at mechanosensitive sites across the membrane

**Figure 2:**
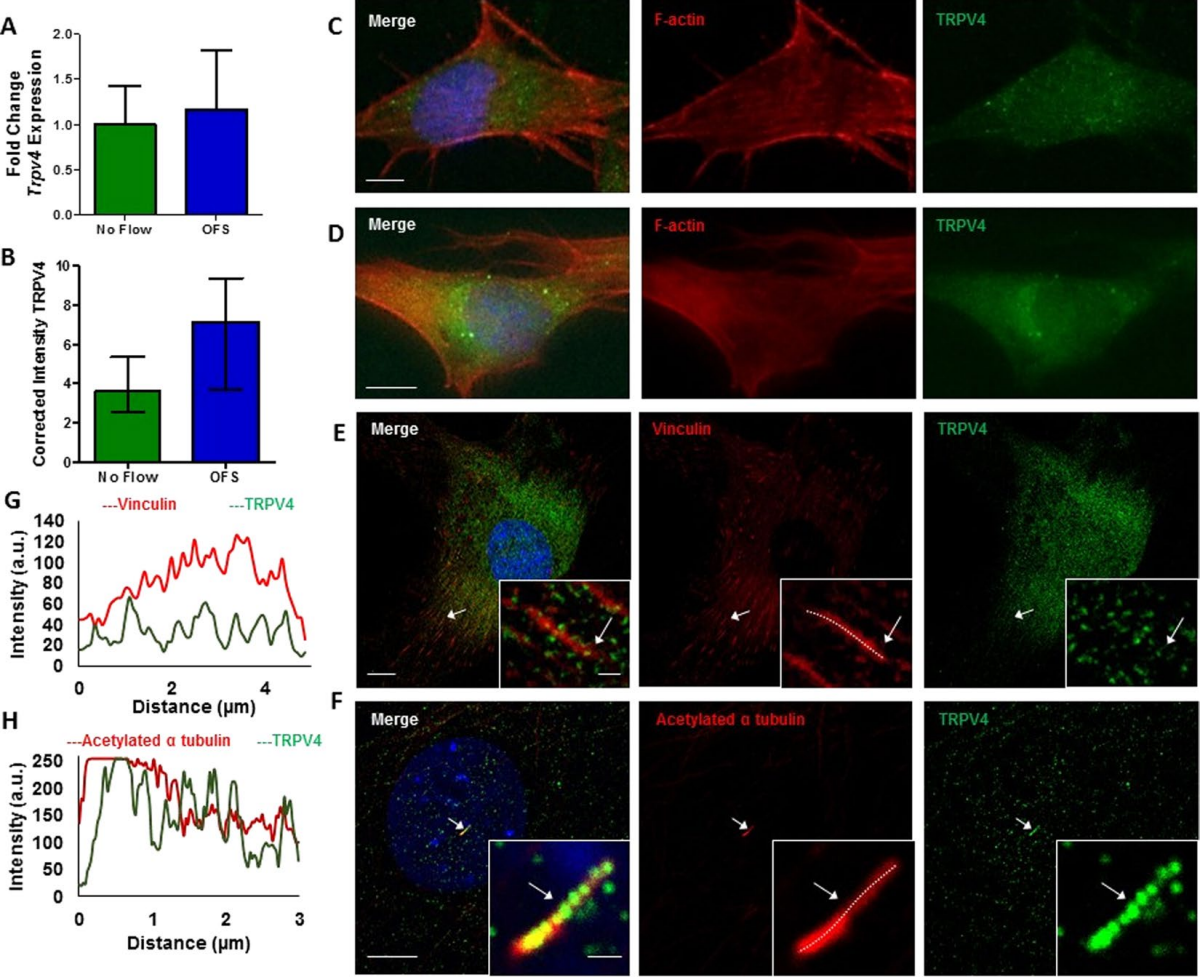
(**A**) *Trpv4* gene expression in cells exposed to 1 Pa 1 Hz OFS for 2 hours, Mean ± SEM N = 3, n = 9–10, student’s t-test, p > 0.05. **(B**) Quantification of cell wide Trpv4 stain intensity under OFS, Median ± Interquartile n = 18–21, Mann Whitney test, **p < 0.01. **(C**,**D**) Immunofluorescent staining of MSCs showing TRPV4 expression and the cytoskeleton (F-actin) (scale bar = 10 µm) following 2 hours at (**C**) static conditions and (**D**) 1 Pa, 1 Hz oscillatory fluid flow. (**E**,**F**) Immunofluorescent staining of MSCs showing TRPV4 expression and **(E**) focal adhesions using vinculin (scale bar = 10 µm, inset 1 µm) and **(F)** primary cilia using acetylated alpha tubulin (scale bar = 10 µm, inset 1 µm). **(G**,**H)** Fluorescence intensity at region marked by arrow, along dashed white line in inset of **(G**) focal adhesion and **(H**) primary cilium.

Given the role of the mechanosensitive calcium channel TRPV4 in cellular mechanotransduction in lineage committed cells, TRPV4 expression in MSCs was determined at the mRNA level using qRT-PCR and at the protein level using immunocytochemistry. Initially, to investigate the expression of TRPV4 and to determine whether TRPV4 is mechanically regulated, MSCs were exposed to 1 Pa OFS for 2hrs. TRPV4 was successfully amplified and there was a non-significant trend of increased expression following mechanical stimulation compared to static controls (p = 0.8254) (Fig. 2A). To further verify these findings at the protein level, immunofluorescence microscopy analysis was performed with antibodies against TRPV4 and F-actin (phalloidin; to detect cell area) following 1 Pa OFS for 2hrs and fluorescence intensity per area was quantified. TRPV4 staining was present across the whole cell area and TRPV4 protein intensity is significantly increased 1.53-fold (n ≥ 18, p < 0.01) in response to OFS demonstrating that the expression of this channel is mechanoregulated (Fig. 2B,C, Supplementary Figure 1). We next examined whether TRPV4 preferentially localizes to sites within the cell which are known to experience high strain under fluid shear and have been previously been shown to play a role in mechanotransduction^38,39^. Therefore, immunofluorescence confocal microscopy was performed in MSCs treated with antibodies against TRPV4, vinculin to identify focal adhesions (FAs) and acetylated-α-tubulin to identify the primary cilium. Vinculin staining clearly identified focal adhesions located along the periphery of the cell as demonstrated in Fig. 2D. TRPV4 staining was punctate throughout the cell area with faint co-localization identified at focal adhesions. This faint co-localization was verified by fluorescent intensity readings along identified FAs (Fig. 2E). Acetylated-α-tubulin staining identified primary cilia as rod-like structures on the apical surface of MSCs located above or adjacent to the nucleus. Unlike at FAs, there was a striking co-localization of TRPV4 along the ciliary axoneme (Fig. 2F), particularly at the base which is predicted to experience the greatest membrane strain under fluid shear^40^. This was further verified by fluorescent intensity (Fig. 2G). This data therefore demonstrates that MSCs express TRPV4 and that this expression is mechanically regulated. Furthermore, TRPV4 is specifically localized to areas within the cell that experiences high strain under fluid shear, particularly at the primary cilium.

### TRPV4 is required for oscillatory fluid shear mediated calcium signaling in MSCs

**Figure 3:**
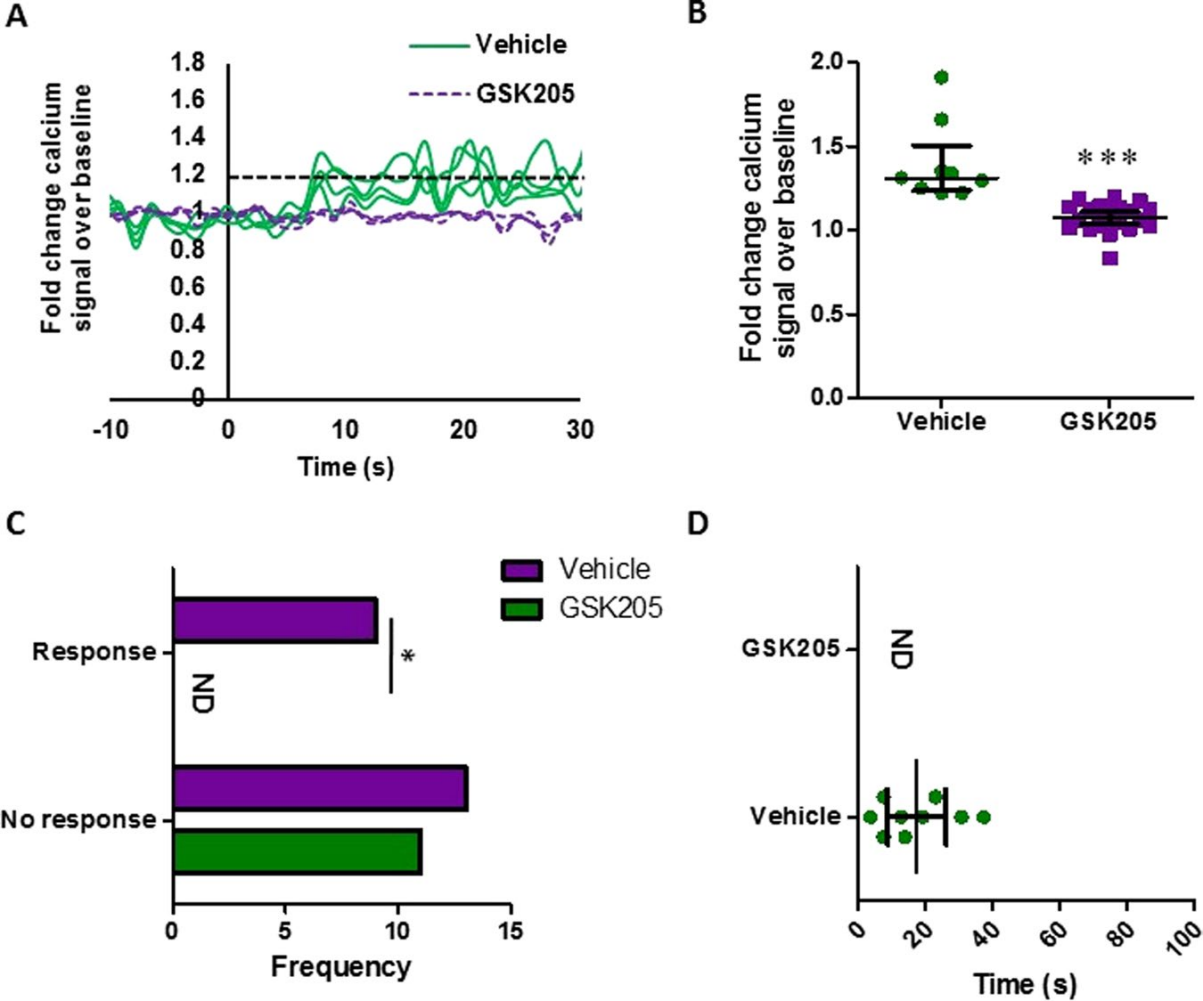
(**A**) Representative calcium profile of individual MSCs responding to OFS generating 1 Pa, 1 Hz. OFS begins at t = 0 s. Cells treated with vehicle alone, DMSO, represented in the solid green; those treated for 1 hour pre mechanical stimulation with 10 µM GSK205 are in the purple dashed line. Baseline values calculated from the 20 seconds prior to the application of OFS. The dashed black line at 1.2-fold change marks the value above which cells are considered to be responsive. **(B)** Median ± Interquartile range fold increase in calcium at first peak following application of treatment. Vehicle: N = 4, n = 9; GSK205: N = 4, n = 22, Mann Whitney test, ***p < 0.001. **(C)** Frequency of cells responding to the stimulus. Median ± Interquartile: Vehicle: N = 4, n = 22; GSK205: N = 4, n = 11, Fisher’s exact test, *p < 0.05. **(D)** Time to first peak for individual cells following application of stimulus at time 0 s. Bars mark Median ± Interquartile, Vehicle: N = 4, n = 9; GSK205: N = 4, n = 22 ND = None determined, no responsive cells observed.

Employing the TRPV4 specific antagonist, GSK205, the role of TRPV4 in oscillatory fluid shear-induced calcium signaling was examined. MSCs were treated with GSK205 or DMSO, vehicle control and exposed to 1 Pa OFS and intracellular calcium levels were analyzed in real-time. Analysis of the calcium signal in MSCs treated with DMSO vehicle control demonstrated a similar response to that shown in Fig. 1A, with OFS inducing rapid calcium increases (17.54 seconds) averaging 1.40-fold in 40% of cells (Fig. 3), demonstrating that DMSO treatment alone does affect mechanically-induced calcium signaling in MSCs. Interestingly, in the absence of TRPV4 functionality, a dramatic loss in calcium signal is observed. No cell met the criteria for mechanoresponsive cells, i.e. greater than 1.2-fold change fluorescence. These cells were responsive to ionomycin, a ionophore applied to the cells to empty stores of calcium and test the functionality of the calcium sensor in each cell. However, the average maximum calcium peak in response to fluid shear was significantly lower than the control group at 1.08 ± 0.02-fold change in the GSK205 group. Therefore, specific antagonization of TRPV4 elicited a complete loss in oscillatory fluid shear induced calcium signaling in MSCs.

### TRPV4 is required for oscillatory fluid shear mediated increases in osteogenic gene expression

**Figure 4:**
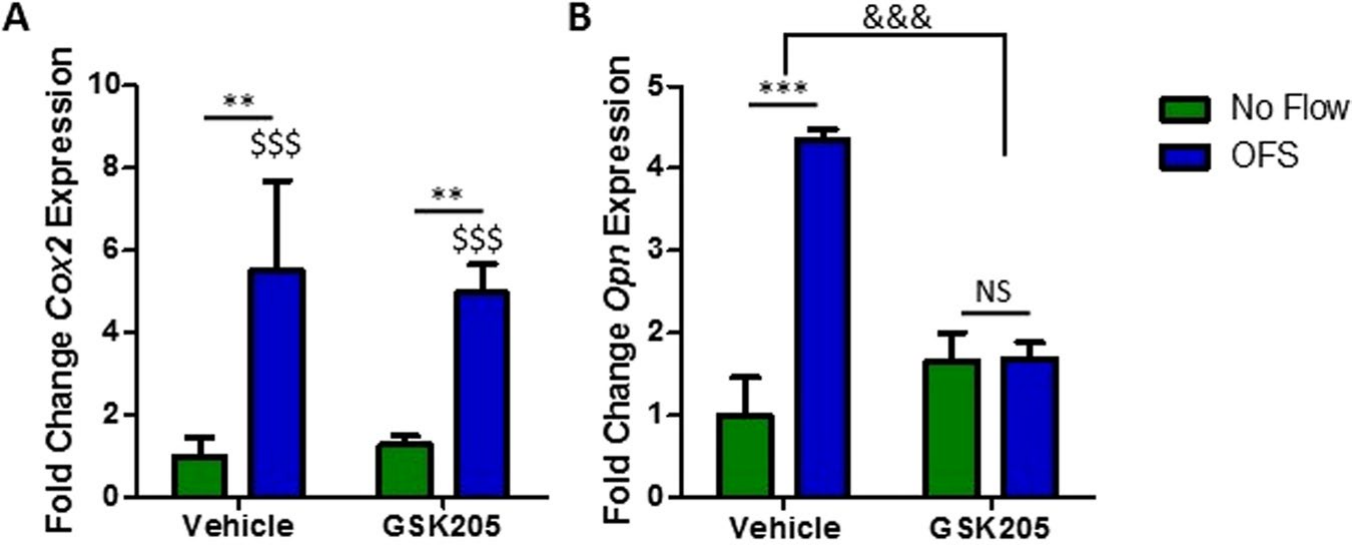
(**A**) *Cox2* and (**B**) *Opn* gene expression in MSCs treated with vehicle control or 10 µM GSK205 for 3 hours following 2 hours at static conditions (green) or 1 Pa 1 Hz OFS (blue), represented as Mean ± SEM, N = 4–6, n = 12–21, 2 way ANOVA with Bonferroni post-hoc test; NS p > 0.05, **p < 0.01, ***p < 0.001 comparison to the no flow vehicle control. ^$$$^p < 0.001 significant effect of OFS on Cox2 expression compared to No flow controls. ^&&&^p < 0.001 significantly different effect of flow stimulus on the vehicle and antagonist treatment groups.

Considering the requirement for TRPV4 in oscillatory shear stress mediated calcium signaling, the role of TRPV4 activity in fluid shear induced gene expression was next investigated. As above, MSCs were treated with GSK205 prior to application of 1 Pa fluid shear oscillating at 1 Hz for 2hrs. *Cox2* and *Opn* in the No Flow control group were unaffected by the antagonist treatment (Fig. 4A,B) demonstrating that TRPV4 activity does not significantly influence basal osteogenic gene expression. Control MSCs treated with DMSO elicited a significant increase in both *Cox2* (5.5-fold, n ≥ 15, p < 0.001) and *Opn* (4.35-fold, n ≥ 14, p < 0.01) when compared to No Flow (Fig. 4A,B), as previously demonstrated in Fig. 1. However, the osteogenic response of MSCs treated with GSK205 following OFS was inhibited, with a reduced increase in *Cox2* (4.97-fold, n ≥ 13, p < 0.01) and a complete loss of the increase in *Opn* (1.69-fold, n ≥ 12) gene expression (Fig. 4A,B). Thus, the mechanosensitive calcium channel TRPV4 is required for oscillatory fluid shear induced increases in early markers of osteogenesis.

### Biochemical activation of TRPV4 induces a calcium response that mimics that produced by oscillatory fluid shear

**Figure 5:**
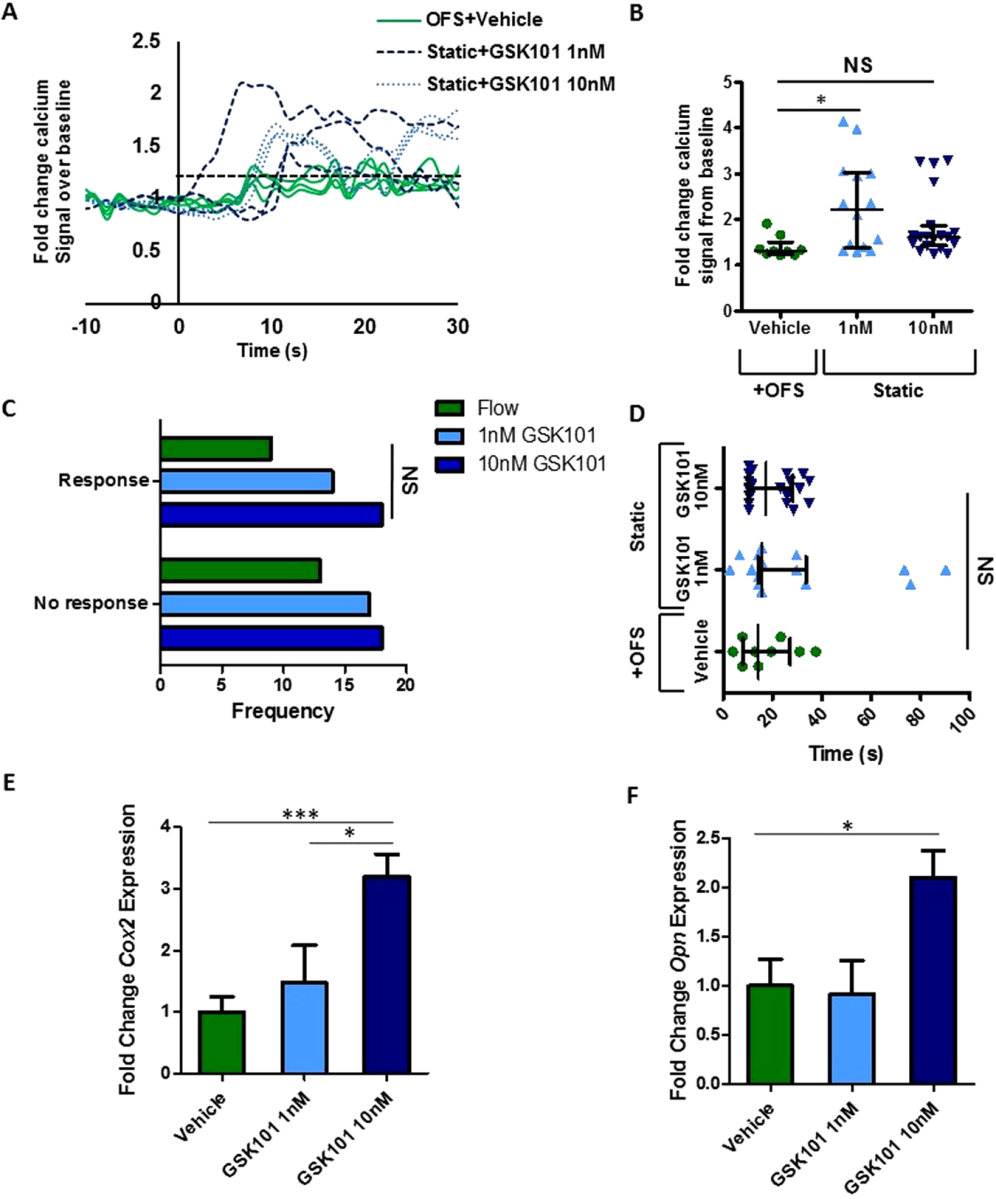
(**A**) Representative calcium profile of individual MSCs responding to OFS generating 1 Pa, 1 Hz shear stress. OFS begins at t = 0 s. Cells treated with equal amounts of the vehicle, DMSO and exposed to OFS are in solid green, 1 nM GSK101 by the dashed blue line and 10 nM GSK101 are represented by the dark blue dotted line. All biochemical treatments are applied only at the onset of fluid shear. Baseline values were calculated from the 20 seconds prior to the application of OFS. The dashed black line at 1.2 fold change marks the value above which cells are considered to be responsive. **(B)** Median and interquartile range of fold increase in calcium at first peak following application of treatment. Control N = 4, n = 9, 1 nM GSK101 N = 4, n = 14, 10 nM GSK101 N = 4, n = 19, Kruskal-Wallis test with Dunn’s multiple comparisons post test, *p < 0.05, NS p > 0.05. **(C**) Frequency of calcium response in MSCs exposed to stimulus, OFS N = 4, n = 9, 1 nM GSK101 N = 4, n = 31, 10 nM GSK101 N = 4, n = 27, Chi-square test, NS p > 0.05. **(D)** Time to first peak for individual cells following application of stimulus at time 0 s. Bars mark Median and interquartile range, Control N = 4, n = 9, 1 nM GSK101 N = 4, n = 14, 10 nM GSK101 N = 4, n = 19. Kruskal Wallis test with Dunn’s post-test, NS p > 0.05. **(E**) *Cox2* and **(F**) *Opn* expression in MSCs treated with DMSO only, Vehicle, 1 nM and 10 nM GSK101 for 2 hours, Mean ± SEM N = 2–3, n = 7–13, 1 way Anova with Bonferroni post-test, *p < 0.05, ***p < 0.001.

Considering the demonstrated role of TRPV4 in MSC mechanotransduction, an approach to target this calcium channel to mimic the effect of OFS was examined using the TRPV4 specific agonist GSK101. Real time analysis of calcium signaling was performed in MSCs treated with increasing concentrations of GSK101 in the place of OFS as in previous experiments. The application of 1 nM and 10 nM GSK101 was promptly marked by a rise in calcium within the cytosol in the absence of mechanical stimulation, at a similar frequency to those subjected to OFS (Fig. 5A,C). Notably, the calcium signal in response to both 1 nM and 10 nM GSK101 is similar to that elicited by OFS in terms of magnitude (1 nM, 2.30-fold; 10 nM, 1.86-fold, Fig. 5B) and time (10 nM, 19.85 ± 2.19 s, Fig. 5D), however the response to 1 nM elicited a more variable response greater than that seen in response to fluid shear (1 nM, 29.52 ± 7.13 s). Overall, the biochemical activation of TRPV4 can induce a calcium signal comparable to that elicited under oscillatory fluid shear.

### TRPV4 activation induces an increase in osteogenic gene expression that mirrors that seen with mechanical stimulation

To further the potential of therapeutically targeting TRPV4 to mimic the effect of OFS, MSCs were treated with GSK101 for 2hrs and osteogenic genes *Cox2* and *Opn* were analyzed as before. Analysis of *Cox2* and *Opn* was carried out using qPCR and fold change was calculated against vehicle treated controls. 1 nM GSK101 treatment resulted in an upward trend, but non-significant increase in *Cox2* (1.14-fold, n ≥ 6, p = 0.7640) and elicited a slight decreasing trend in *Opn* gene expression (0.80-fold, n ≥ 7, p = 0.5548) (Fig. 5E,F). However, 10 nM GSK101 treatment induced a significant 2.49-fold increase in *Cox2* (n ≥ 8, p < 0.01) and a 1.83-fold increase in *Opn* (n ≥ 7, p < 0.05). Both osteogenic responses with 10 nM treatment compare favorably to that elicited in response to OFS (Fig. 1E,F), demonstrating that GSK101 can be utilized to therapeutically mimic the anabolic effect of mechanical loading.

### Biochemical modulation of TRPV4 activity influences bone matrix deposition

**Figure 6:**
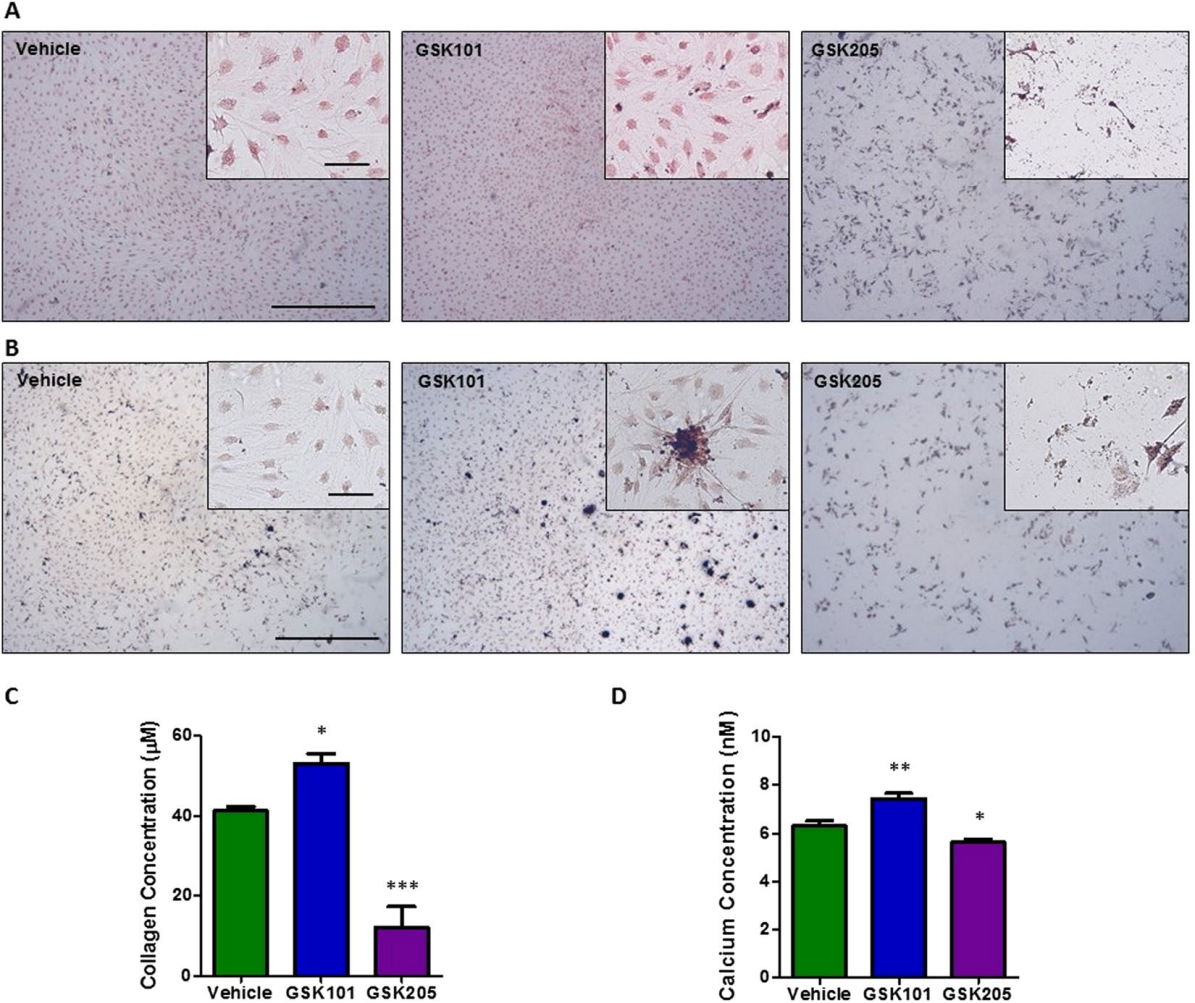
Representative images of **(A)** picrosirius red and **(B)** alizarin red s staining of MSCs following 21 days treatment with vehicle (DMSO), GSK101 and GSK205 at 2× (scale bar = 500 µm), inset at 10×, (scale bar = 100 µm). Quantification of **(C)** picrosirius (collagen) and **(D)** alizarin (calcium) stains extracted from each group in **(A)** and **(B)** n = 6, statistical analysis One-way ANOVA with Dunnett’s multiple comparisons post test *p < 0.05, **p < 0.01, p < 0.001.

Given the demonstrated role of TRPV4 in mediating early biophysical and biochemical osteogenic responses in MSCs, we next investigated whether long term targeting of this channel could directly influence full osteogenic lineage commitment of progenitor cells. Therefore, MSCs were cultured with the TRPV4 antagonist GSK205 or agonist GSK101 over a 21 day period and bone matrix deposition in terms of collagen and calcium deposition was analysed. After 21 days of TRPV4 inhibition, GSK205 significantly inhibited the deposition of collagen and mineral by MSCs (0.28-fold Picrosirius, n = 6, p < 0.001 and 0.89 fold Alizarin, n = 6, p < 0.05) (Fig. 6C). Moreover, significant cell death was evident in this group indicating a role for TRPV4 in cell viability. Interestingly, similar to that seen at early timepoints, TRPV4 activation over 21days resulted in a significant increase in collagen and mineral deposition (1.29-fold Picrosirius, n = 6, p < 0.05, 1.18-fold Alizarin, n = 6, p < 0.01) (Fig. 6D) further supporting the early evidence for the positive influence of GSK101 on osteogenesis and confirms a role for TRPV4 in the osteogenic differentiation of MSCs.

### Mesenchymal stem cells with defective intraflagellar transport display an altered response to TRPV4 activation

**Figure 7:**
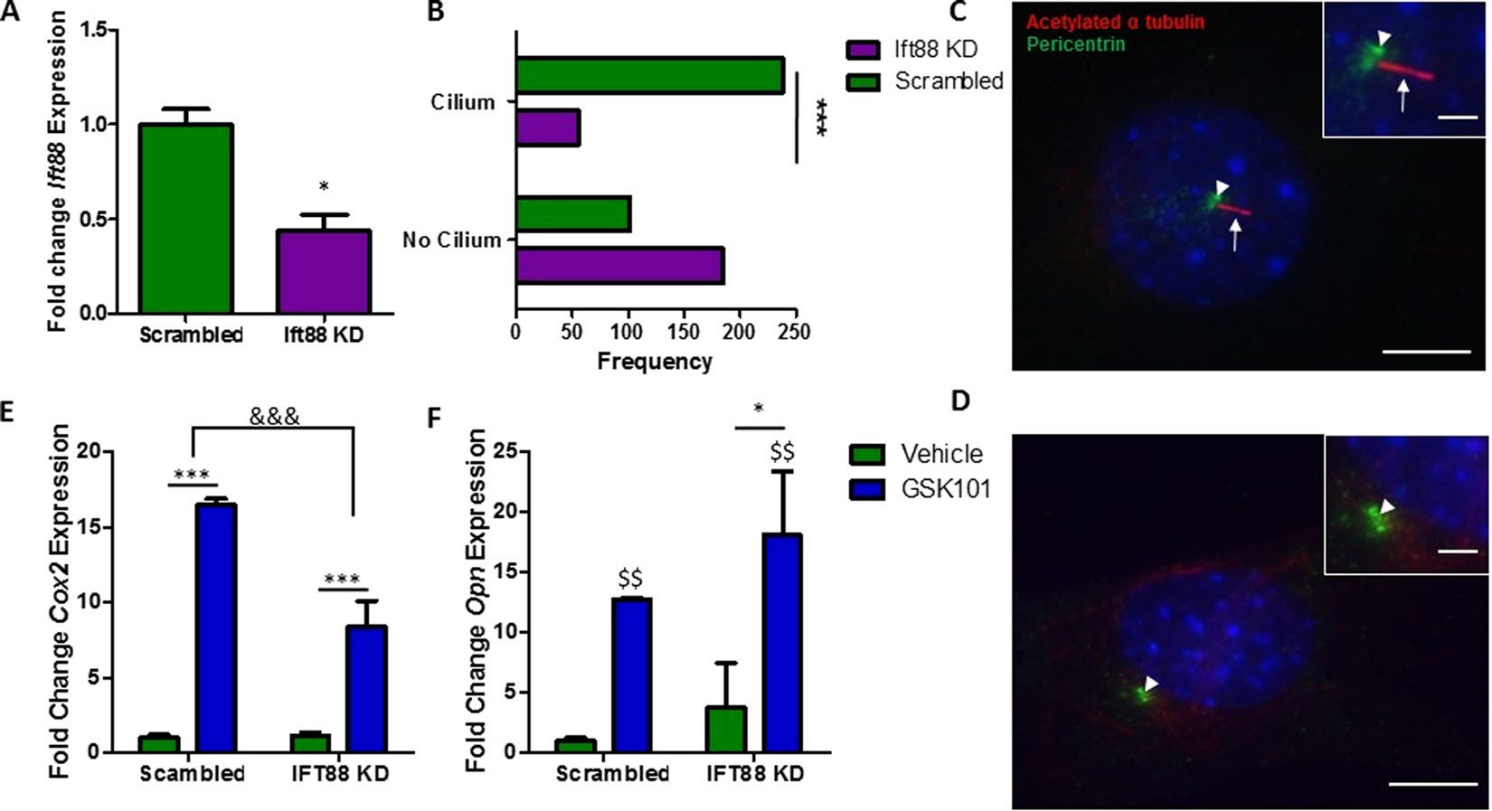
(**A**) Validation of inhibition of *Ift88* gene expression, N = 4, student’s t-test with Welch’s correction, *p < 0.05. **(B)** Frequency of primary cilium formation following transfection with siRNA, N = 4–6, n = 286–293, Fisher’s exact test, ***p < 0.001. **(C**,**D)** Immunostaining of cells transfected with either off target control **(C**) or *Ift88* targeted siRNA **(D**) stained with acetylated α-tubulin, (primary cilium, red; arrows) and pericentrin (centrioles, green; arrow head). Nuclei are counterstained with DAPI (blue). Scale bars represent 5μm and 1μm (insert). **(E**) *Cox2* and **(F**) *Opn* gene expression in MSCs transfected with siRNA targeting *Ift88* or scrambled control, following 2 hours of treatment with 10 nM GSK101 or vehicle control, represented as Mean ± SEM, N = 3, n = 5–8, 2 way ANOVA with Bonferroni post-test, *p < 0.05, ***p < 0.001 significant in comparison to vehicle control, ^$$^p < 0.01 significant effect of GSK101 treatment, ^&&&^p < 0.001 significant effect of the treatment on the response to GSK101.

Given the localization of TRPV4 to the primary cilium, a known site of mechanotransduction, the role of the cilium in TRPV4-mediated osteogenic responses was further investigated. The formation of cilia was inhibited through the utilization of siRNA targeting IFT88. IFT88 is a principal motor protein required for ciliogenesis. The transfection resulted in significantly diminished *Ift88* mRNA expression and the frequency of primary cilia was significantly reduced as demonstrated by immunocytochemistry (Fig. 7A–D). In the remaining cells, which still possessed a cilium, the axoneme length was stunted which is suggestive of defective IFT transport and cilia function. MSCs transfected with siRNA targeting IFT88 (cilium knockdown) or scrambled siRNA were treated with 10 nM GSK101 for 2 hours and the expression of osteogenic genes *Cox2* and *Opn* was examined. Basal levels of *Cox2* and *Opn* were not significantly changed in MSCs which do not possess a primary cilium (Fig. 7E,F), when compared to control MSCs. TRPV4 activation via GSK101 treatment in MSCs transfected with scrambled control elicited a significant increase in *Cox2* (16.5-fold, n ≥ 5, p < 0.001) and *Opn* (12.7-fold, n ≥ 5, p < 0.001). However, in MSCs which do not possess a primary cilium, TRPV4 mediated increases in *Cox2* gene expression are significantly less than off target siRNA transfected controls (Fig. 7E), suggesting that TRPV4 localization to the cilium is functionally important in terms of eliciting a *Cox2* response. Interestingly, deletion of IFT88 resulted in a slight but significant increase in the TRPV4-mediated *Opn* response (p < 0.05) (Fig. 7F). Together, this data indicates that TRPV4 localization to the primary cilium is functionally important but the significance is potentially pathway dependent.

## Discussion

A potent regulator of skeletal health is physical loading; with the application of an external mechanical stimulus known to enhance MSC osteogenesis and bone formation^3,41^. Therefore, understanding the mechanisms by which physical loading can drive stem cell osteogenic differentiation is pivotal to the development of novel anabolic therapies to promote bone formation in diseases such as osteoporosis through the targeting of this progenitor population^42^. In this study, we demonstrate that TRPV4 is a key component of MSC mechanotransduction, regulating oscillatory fluid shear-induced calcium signaling and osteogenic gene expression. Critically, we also demonstrate that TRPV4 can be targeted pharmacologically, eliciting an anabolic response that mirrors that seen with mechanical stimulation, demonstrating mechanotherapy potential. Interestingly, we also show that this mechanosensitive channel localizes to the primary cilium, a known site of MSC mechanotransduction^13^, and demonstrate that the primary cilium is in part required for the TRPV4-mediated increases in *Cox2*. In summary, this data demonstrates a novel mechanism of stem cell mechanotransduction, which can be targeted therapeutically, and further highlights the critical role of the primary cilium in stem cell biology.

TRPV4 mediates OFS mechanotransduction in MSCs. By mimicking the marrow mechanical environment through the application of OFS, this study demonstrates that MSCs transduce a mechanical stimulus rapidly, resulting in a 1.53-fold increase in cytosolic calcium. This response is consistent with previous work in cells of the osteogenic lineage in response to OFS^9,16,24,43^, and demonstrates the utilization of calcium signaling as an important second messenger in MSC mechanotransduction. Interestingly, the complete loss of calcium signaling in MSCs treated with GSK205, an antagonist selective for TRPV4, suggests that TRPV4 is the principal mediator of calcium responsive mechanotransduction in MSCs. This finding is consistent with recent studies comparing the role of TRPV1 and TRPV4^43^. Previous studies in chondrocytes, endothelial cells and osteocytes demonstrate a similar dependence on TRPV4 in terms of loading induced calcium signaling^31,33,44^, highlighting the role of this channel in the mechanobiology of multiple tissues in addition to progenitors. Interestingly, the inhibition of TRPV4 also diminished the osteogenic response to shear in terms of *Cox2* and *Opn* gene expression. *Cox2* is required for the production of PGE_2_ and has roles in MSC osteogenesis, loading-induced bone formation and fracture repair^45,46^. Although increases in *Cox2* displayed a decreasing trend following TRPV4 inhibition, a significant increase in *Cox2* was still achieved in response to OFS. Therefore, although TRPV4 plays a role in *Cox2* responses, alternate mechanotransduction mechanisms may compensate for the loss of TRPV4. For example, OFS-induced increases in *Cox2*, which have previously been linked to calcium signaling, have also been shown to be dependent on cAMP signaling^47^, and thus may be activated by multiple mechanisms. *Opn* has demonstrated diverse functions in osteogenic cells, acting as a chemoattractant and regulator of proliferation and mineralization^48–50^. The complete loss of the *Opn* response to OFS following TRPV4 inhibition is consistent with increases in *Opn* being calcium dependent^16^. A role for TRPV4 in modulating gene expression in response to mechanical load is similar to that demonstrated in chondrocytes^31^ and osteocytes^24^ demonstrating that TRPV4 is an important channel mediating mechanotransduction in progenitor cells through to lineage committed populations. Furthermore, modulation of TRPV4 activity effects the osteogenic differentiation of MSCs over a 21 day culture period. Inhibition of TRPV4 activity in long term culture is detrimental to cell health and this substantiates the importance of its role in cell survival and differentiation. In summary, TRPV4 is a critical component of MSC mechanotransduction and thus identifies this channel as a potential target to mimic the pro-anabolic effect of loading.

Biochemical activation of TRPV4 elicits a calcium second messenger and osteogenic response that mirrors that seen with oscillatory fluid shear in MSCs. The TRPV4 specific agonist GSK101, at either 1 nM or 10 nM, induced a calcium response of similar magnitude and timing to that elicited by oscillatory fluid shear. However, 1 nM GSK101 elicited a more variable calcium response in terms of magnitude and time to peak suggesting that this is approaching the lower threshold for robust TRPV4 activation in MSCs. Furthermore, 1 nM GSK101 did not induce an osteogenic response, but a significant increase in *Cox2* and *Opn* was achieved following 10 nM treatment. This induced mechanoresponse is consistent with previous work by O’Conor *et al*. which demonstrated that GSK101 elicits an increase in calcium and gene expression in chondrocytes, mirroring that seen with dynamic loading^31^. While TRPV4 activation in chondrocytes promotes cartilage matrix deposition, in progenitor cells we have found GSK101 treatment increases the collagen and calcium produced by MSCs over a 21-day period and this influence on the osteogenic commitment of MSCs confirms the significance of the early gene expression changes in lineage commitment downstream. Together with our observations of an early anabolic response mediated by TRPV4 activation, this demonstrates that TRPV4 can be targeted therapeutically to elicit an osteogenic response that mimics that seen with physical loading.

TRPV4 is a mechanoregulated channel that specifically co-localizes to the primary cilium; an organelle which experiences high strain under fluid shear and is a known site of stem cell mechanotransduction^13^. Despite previous evidence for TRPV4 co-localization with integrins in endothelial cells^29,33^, TRPV4 was only found to co-localize to the primary cilium, with intense staining evident towards the ciliary base. Interestingly, under fluid shear the deflection of the ciliary axoneme results in a concentration of strain at the base which may be sufficient to directly stretch open this channel^40,51–53^. This specific localization to areas of high strain may potentially increase the mechanosensitivity of the cell. Furthermore, due to the discrete cylindrical microdomain of the cilium and the specific localization of a plethora of signaling molecules, localization of TRPV4 to this signaling center may amplify and enhance the rate of TRPV4-mediated mechanosignaling^54^. This spatial organization of TRPV4 is consistent with observations in lineage committed cells such as osteocytes, chondrocytes and cells of the endothelium and the trabecular meshwork of the eye^24,34,55,56^. Intriguingly, in MSCs which do not possess a primary cilium, TRPV4 mediated increases in *Cox2* gene expression are decreased, indicating that the localization of TRPV4 to the cilium is functionally significant. This loss in TRPV4-mediated Cox2 upregulation in MSCs following cilia removal is consistent with the loss in OFS-mediated Cox2 upregulation in MSCs which do not possess a primary cilium^13^. Therefore, the mechanism of cilia-mediated MSC mechanotransduction may include TRPV4. Furthermore, chemical removal of primary cilia from chondrocytes, using chloral hydrate, leads to a significant reduction in TRPV4-mediated calcium signaling but does not inhibit the calcium response to ionomycin indicating a similar role for the cilium localized TRPV4 in cartilage^34^. Interestingly, the loss of the cilium did not prevent the TRPV4-mediated increase in *Opn* gene expression but augmented it, suggesting that *Cox2* and *Opn* may be activated via alternate pathways downstream of TRPV4, with TRPV4-mediated *Opn* regulation being independent of the primary cilium. The increase in the *Opn* response in the presence of defective cilia indicates that this alternative pathway may be compensating for the loss in the *Cox2* response. In summary, the co-localization of TRPV4 to the primary cilium is significant and highlights the important role of the cilium in MSC biology.

## Conclusion

This study presents evidence for the role of TRPV4 in MSC mechanotransduction, presenting a novel mechanism by which oscillatory fluid shear is transduced into a biochemical bone forming response in this progenitor population. This therefore highlights this channel as a therapeutic target and we have demonstrated that TRPV4 can be activated pharmacologically mimicking the anabolic effect of loading by activating second messenger calcium signaling, osteogenic gene expression and bone matrix deposition demonstrating mechanotherapeutic potential. Lastly, we demonstrate that the cilium plays a critical role in TRPV4-mediated signaling in MSCs likely through the co-localization of this channel to the microdomain of this mechanosensory organelle.

## Methods

### Mesenchymal Stem Cell Culture

The murine mesenchymal stem cell line C3H10T1/2 was obtained from ATCC (LGC Standards, Teddington, Middlesex, UK, http://www.lgcstandards-atcc.org). MSCs were maintained in Dulbecco’s modified Eagle’s medium (DMEM) with low glucose (Sigma-Aldrich Ireland Ltd. Arklow, Ireland, http://www.sigmaaldrich.com/ireland.html) supplemented with 10% fetal bovine serum (FBS) (South American origin, Labtech International, Ltd. Heathfield, East Sussex, UK) and 1% Penicillin Streptomycin (P/S) (Sigma). This cell line has previously been shown to undergo both biochemical and biophysically induced osteogenic lineage commitment^3^. For extracellular matrix formation MSCs were supplemented with 10 nM Dexamethasone (Sigma), 10 mM β-glycerophosphate (Sigma) and 0.05 mM Ascorbic acid (Sigma) to provide the components necessary for osteogenic matrix formation.

### Gene expression

TRI reagent (Sigma) was used to extract RNA per the manufacturer’s protocol. The concentration of RNA in each sample was measured using a Nanodrop spectrophotometer and sample purity was checked via 260/280 and 260/230 absorbance ratios. 100–800 ng of RNA was reverse transcribed to cDNA using the High-Capacity cDNA Reverse Transcription Kit (Applied Biosystems, Foster City, CA, USA, https://www.thermofisher.com). Quantitative polymerase chain reactions (qPCR) were prepared for all samples using SYBR Select Master Mix with ROX passive dye (Applied Biosystems, 4472903) and custom designed primers (Sigma) for *18 s, Cox2* and *Opn*. *TRPV4* qPCR reactions were prepared using TaqMan Universal PCR Mix (Applied Biosystems, 4304437) with ROX passive dye and a pre-designed Taqman Gene Expression Assay (Applied Biosystems, 4331182) as outlined in Supplementary Table 1 for amplification using the ABI 7500 real time PCR machine (Applied Biosystems). The relative quantity of each sample was calculated with reference to *18 s* and expressed as fold change normalized to the control group.

### Parallel Plate Flow Chambers

Parallel plate flow chambers were designed in house as described previously^3^. Briefly, MSCs were seeded on fibronectin (10 µg/ml) coated glass slides, assembled between two plates and attached to a programmable syringe pump (New Era Pump Systems Inc. Farmingdale, NY, USA, http://www.syringepump.com). Oscillating fluid shear (OFS) was applied through a 10 ml syringe (Becton Dickinson and Company, Franklin Lakes, NJ, USA, www.bd.com) at 52.5 ml/min and at a frequency of 1 Hz subjecting cells to a shear stress of 1 Pa. The MSCs were lysed for mRNA extraction or fixed for immunocytochemical staining immediately after the application of fluid shear for 2 hours. The no flow controls were similarly assembled within the chambers but were not subjected to fluid shear.

### Calcium Imaging

Changes in calcium signaling were observed using the cell permeant calcium indicator Oregon Green 488 BAPTA-1AM (OGB) (Invitrogen, Carlsbad, CA, USA, https://www.thermofisher.com). OGB stock was prepared in DMSO at 2 mM, all further dilutions were in phenol red free DMEM, 0.5% FBS. MSCs were seeded on fibronectin (Sigma) coated glass slides (No. 2- CN Technical, Wisbech, UK, http://www.cntech.co.uk) at a density of 3400/cm^2^. After 48 hours in culture cells were incubated in 10 µM OGB at room temperature for 45 minutes before rinsing twice in phosphate buffered saline (PBS) (Sigma). Slides were assembled in a parallel plate flow chamber (RC30- Warner Instruments, Hamden, CT, USA, https://www.warneronline.com) in phenol red free DMEM (Sigma), 0.5% FBS, and incubated for a further 15 minutes. Initially medium was perfused through the chamber at 0.028 ml/min (0.01 Pa shear stress) (New Era) for 2 minutes, followed in the case of OFS treatment by 2.8 ml/min (1 Pa) oscillating across the chamber at 1 Hz for 5 minutes. The indicator was imaged using an Olympus IX83 epifluorescent microscope (Olympus, Hamburg, Germany https://www.olympus-europa.com/) at 40× (N.A. 0.60 Air). Exposure time was kept below 600 ms and was kept constant between control and treatment groups, allowing image acquisition every 1.29 s. Following each experiment, 10 µM ionomycin (Alomone Labs, Jerusalem, Israel, http://www.alomone.com) was applied to the cells as a positive control for sensitivity of the indicator to calcium. Response was calculated as fold change fluorescence over baseline levels where baseline was taken as the 30 seconds prior to treatment (t = 0). Cells were only considered responsive if displaying a fold change over baseline greater than 1.2 in response to treatment as well as a greater than 1.2-fold change in calcium signal upon application of ionomycin. Ionomycin was only applied after TRPV4 modulators or biophysical stimuli to validate the data collected and demonstrate the efficacy of the calcium indicator within a given cell. The peak is defined as the apex of the first fluctuation above 1.2 fold change fluorescence in responding cells. In Figs 1B and 3B the fold change magnitude is taken from the highest fluctuation above baseline observed for comparison to the treatments plotted. Further details on calcium quantification can be found in Supplementary Figure 2.

### Immunocytochemistry

MSCs were seeded on fibronectin coated glass coverslips for 24 hours before serum starvation in DMEM low glucose, 0.5% FBS, 1% P/S for 48 hours. After fixation in neutral buffered formalin for 10 minutes (Sigma), coverslips were permeabilized in 0.1% Triton X-100 and non-specific binding sites were blocked using 1% w/v BSA (Sigma) in PBS for 2 hours at room temperature. The primary antibodies targeting the primary cilium (acetylated α tubulin, ab24610, Abcam, Cambridge, UK, http://www.abcam.com) or vinculin (ab18058, Abcam) were applied overnight at 4 °C, diluted 1:1500 and 1:1000 respectively. Next, primary antibodies targeting TRPV4 (TRPV4, ab74738, Abcam) or centrioles (pericentrin, ab448, abcam) were applied for 1 hour at room temperature at a dilution of 1:1000 and followed by an Alexa Fluor 594 conjugated secondary antibody (A21203, Life Technologies, Carlsbad, CA, USA, https://www.thermofisher.com) for acetylated alpha tubulin and vinculin and an Alexa Fluor 488 conjugated secondary antibody (A11008, Life Technologies) for TRPV4 and pericentrin at 1:500. Finally, DAPI (Sigma) was applied for 5 minutes in PBS prior to sample mounting on glass slides using Prolong gold mounting medium (Invitrogen). Imaging was performed on an Olympus IX83 epifluorescent microscope with a 100 W halogen lamp at 100× (N.A. 1.40 Oil)(Fig. 2C,D) or the Leica SP7 (Leica Microsystems, Wetzlar, Germany, http://www.leica-microsystems.com) scanning confocal microscope at 63× (N.A. 1.40 Oil) (Fig. 2E and F). For imaging the primary cilium, the confocal was set to a pinhole of 67 µm and 701.33mAU. The focal adhesions were acquired using settings of 95.6 µm pinhole size and 1.00 AU. Controls in the absence of primary antibody were used to test for non-specific binding and background staining of the secondary antibodies. The line profile tool in LAS X software (Leica) was used to measure the intensity of each channel along manually defined regions of interest. For Fig. 2B the intensity was measured from the area of the cell using phalloidin staining to define the perimeter. Mean fluorescence intensity was corrected for background, the mean intensity in an identical area outside the cell. The median of these corrected values for each treatment, no flow and flow, were compared.

### Biochemical targeting of TRPV4

The TRPV4 channel was inhibited via application of GSK205 antagonist (Merck Millipore, Billerica, MA, USA, http://www.merckmillipore.com) at 10 µM diluted in cell culture medium. Cells were treated with GSK205 supplemented medium for 1 hour prior to application of mechanical stimulation and controls were incubated for the same time frame for calcium signaling and gene expression. TRPV4 was activated via the specific agonist GSK1016790A (Sigma). Cells were not pre-treated with GSK101; supplemented medium was only applied for the duration of the 1 Pa mechanical stimulus. Vehicle controls consisted of <0.1% DMSO for each set up. For long term cell culture GSK205, GSK101 or DMSO were applied at the same concentrations and maintained throughout the culture period via supplementation of the medium which was changed every 3 days.

### Extracellular matrix stain and extraction

To investigate extracellular matrix formation, cells were fixed in formalin following 21 days in culture. Collagen deposition was stained using 1% Picrosirius Red (Sigma) under gentle agitation at room temperature, after 1 hour all wells were rinsed twice in 0.5% acetic acid and distilled H_2_O. Calcium staining was performed using Alizarin Red S at 1% for 20 minutes at room temperature and rinsed in distilled H_2_O until the background was clear of stain. Images were acquired using 2× (NA 0.06) and 10× (NA 0.25) objectives. Collagen deposition was quantified by scraping picrosirius stain from each well in PBS and centrifuging at 14000 g for 10 minutes. The pellet collected was dissolved in 0.5 M NaOH and absorbance measured at 550 nm. Calcium stain was extracted via incubation in 10% acetic acid for 30 minutes under gentle agitation followed by heating at 85 °C for 10 minutes. Cell debris was collected by centrifugation at 20,000 g for 15 minutes and the pH adjusted to pH4.1-4.5 before measuring absorbance at 405 nm.

### Inhibition of primary cilia formation

Intraflagellar transport protein 88 (IFT88) is a protein required for functional ciliogenesis and was targeted with siRNA. Lipofectamine RNAiMAX (Invitrogen) was diluted 1/135 in OptiMEM (Gibco, Foster City, CA, USA, https://www.thermofisher.com) reduced serum transfection medium. This was mixed 1:1 with predesigned Stealth RNAi siRNA targeting IFT88 (MSS211714, Invitrogen) at a dilution of 60 nM in OptiMEM and incubated at room temperature for 15 minutes before application. The off-target control was Stealth RNAi siRNA Negative Control, Medium GC (12935300, Invitrogen). After 24 hours, additional medium was added to each transfection. 48 hours following transfection the transfected cells were seeded for experimentation in DMEM (0.5% FBS, 1% P/S). Cells were cultured for an additional 24 hours before exposure to GSK101. Validation of the efficiency of siRNA knockdown was analyzed at the mRNA level using qPCR and at the protein level using immunocytochemistry (ICC) as described above.

### Data analysis

The relative expression of each gene with reference to *18 s* was calculated and the results expressed as fold change gene expression relative to the control group along with the standard error of the mean. For all gene expression and stain extraction an unpaired two-tailed t-test (α = 0.05) was used to analyze control to treatment conditions or in the case of more than one condition a one-way ANOVA and under multiple conditions a two-way ANOVA were used with a Bonferroni post-test (α = 0.05). For fluorescence intensity measurements, the data sets did not follow a normal distribution and were tested via a Mann Whitney test except in the case of more than one treatment condition where a Kruskal Wallis test was used with Dunn’s post-test. The frequency of MSCs eliciting a calcium response to different stimuli was tested via a Fisher’s exact test for comparison of two treatments or a Chi-square group for multiple treatments. The influence of OFS and GSK205 on gene expression was analyzed via a two-way ANOVA with Bonferroni post-tests (α = 0.05). Stringent parameters identifying a responsive cell in terms of calcium signaling, were defined based on the effect of the ionophore, ionomycin, on the activity of the indicator and the change in fluorescence seen in similar studies of osteoblasts, osteocytes and kidney collecting duct cells^57–60^.

## Electronic supplementary material

Supplementary Information
