# Supplementary material for: TRPV4-mediates oscillatory fluid shear mechanotransduction in mesenchymal stem cells in part via the primary cilium
Source: Sci Rep. 2018 Feb 28;8:3824. doi: 10.1038/s41598-018-22174-3 (PMC5830574; doi:10.1038/s41598-018-22174-3)
Supplement: Supplementary file 1 — Supplementary Information [file 41598_2018_22174_MOESM1_ESM.pdf]

TRPV4-mediates oscillatory fluid shear mechanotransduction in mesenchymal stem cells in part via the primary cilium

Michele A. Corrigan[1,2,3], Gillian P. Johnson[1,2,3], Elena Stavenschi[1,2,3], Mathieu Riffault[1,2,4], Marie-Noelle Labour[1,2,3], David A. Hoey[1,2,3,4].

[1]Trinity Centre for Bioengineering, Trinity Biomedical Sciences Institute, Trinity College Dublin, Dublin 2, Ireland.

[2]Dept. of Mechanical and Manufacturing Engineering, School of Engineering, Trinity College Dublin, Dublin 2, Ireland.

[3]Dept. of Mechanical, Aeronautical and Biomedical Engineering, University of Limerick, Ireland.

[4]Advanced Materials and Bioengineering Research Centre, Trinity College Dublin & RCSI, Dublin 2, Ireland.

*Corresponding Author: Prof. David A. Hoey, Tel: +353 1896 1359, email: dahoey@tcd.ie

Supplementary Figures:

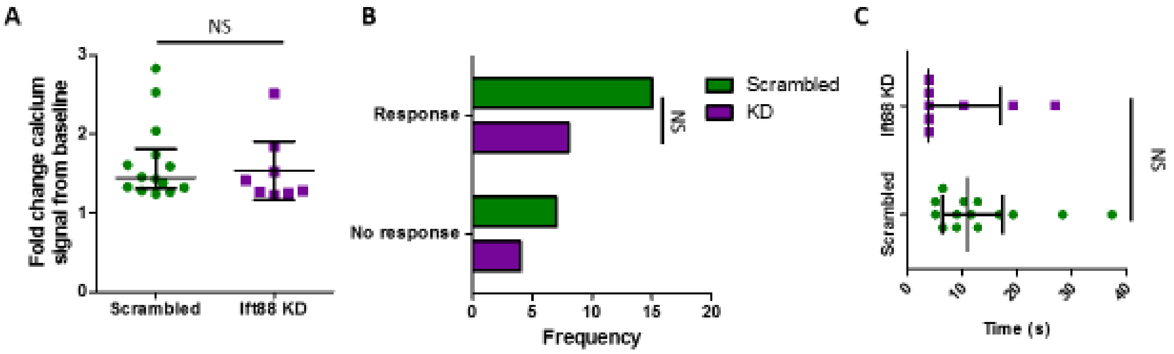

Supplementary figure 1: (a) Median and interquartile range of fold increase in calcium at first peak following application of GSK101 in cells transfected with scrambled siRNA or siRNA targeting IFT88, Mann Whitney test, NS p>0.05. **(b)** Frequency of calcium response in MSCs exposed to GSK101, Fisher's exact test, NS p>0.05. **(c)** Time to first peak for individual cells following application of GSK101 at time 0s. Bars mark Median and interquartile range, Scrambled N=4, n= 20, IFT88 KD N=4, n=12, Mann Whitney test, NS p>0.05.

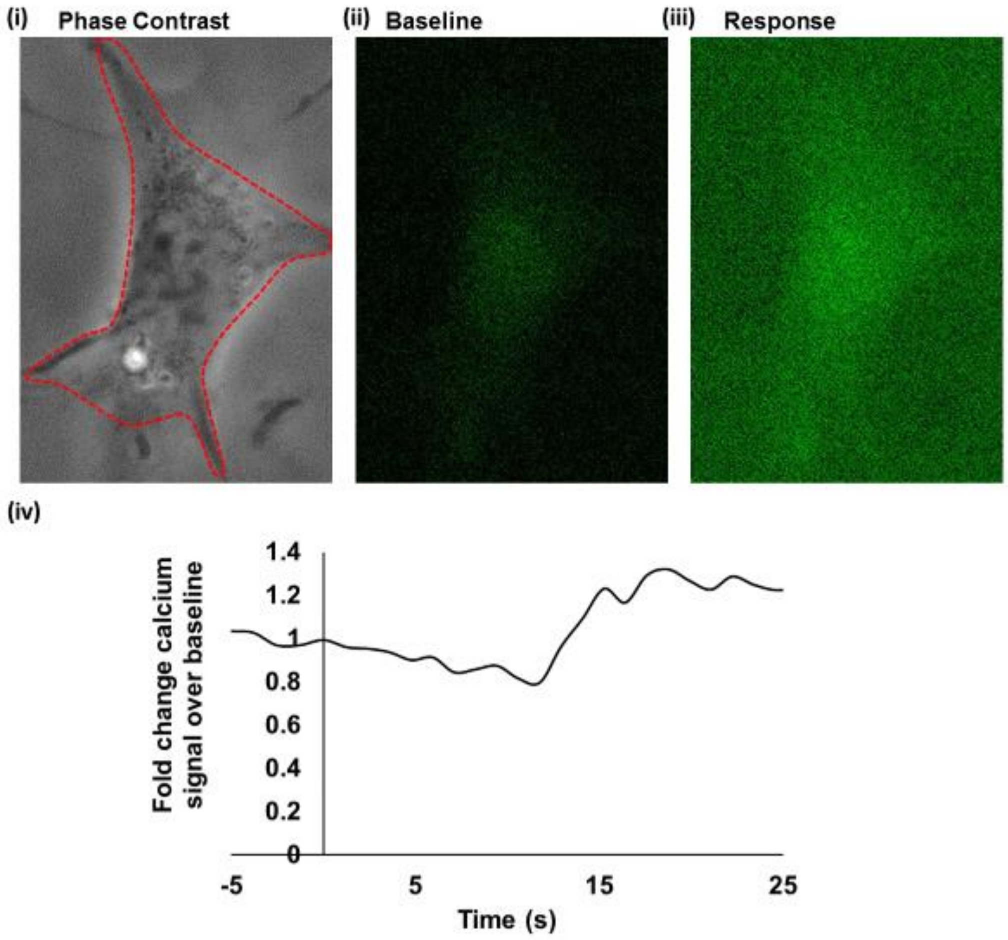

Supplementary Figure 2: Representative images of MSCs during calcium peak. **(i)** Phase contrast image of MSC at 40x (NA 0.6), red line marks the region where intensity is measured. **(ii) (iii)** Fluorescent image of same cell under 488nm filter, **(ii)** before calcium peak and **(iii)** at peak calcium. **(iv)** Trace of fluorescence signal for the cell in (i)-(iii) over time normalized to baseline control and corrected for background fluorescence.

Supplementary Table 1: Primer sequences and concentrations employed in quantitative PCR analysis.

Gene	Tm (°C)	Primer concentration	Sequence	Amplicon Size
18S	60	400 nM	5'-GTAACCCGTTGAACCCCATT-3'	151bp
			3'-CCATCCAATCGGTAGTAGCG-5'	
Cox2	60	400 nM	5'-ACTCATAGGAGAGACTATCAAG-3'	147bp
			3'-GAGTGTGTTGAATTCAGAGG-5'	
Opn	60	400 nM	5'-GGATGAATCTGACGAATCTC-3'	188bp
			3'-GCATCAGGATACTGTTCATC-5'	
TRPV4	60	1x	Mm00499025_m1	56bp
